# Synthesis, antioxidant activity, molecular docking and ADME studies of novel pyrrole-benzimidazole derivatives

**DOI:** 10.55730/1300-0527.3377

**Published:** 2021-02-20

**Authors:** Fikriye ZENGİN KARADAYI, Rahman BAŞARAN, Mehmet Murat KIŞLA, Binay CAN EKE, Zeynep ATEŞ ALAGÖZ

**Affiliations:** 1 Department of Pharmaceutical Chemistry, Faculty of Pharmacy, Ankara University, Ankara, Turkey; 2 Department of Pharmaceutical Toxicology, Faculty of Pharmacy, Ankara University, Ankara, Turkey

**Keywords:** Synthesis, antioxidant activity, pyrrole-benzimidazole, molecular docking, lipid peroxidation

## Abstract

Several 5-(alkylsulfonyl)-1-substituted-2-(1H-pyrrol-2-yl)-1H-benzo[d]imidazole derivatives were synthesized and their antioxidant activities were investigated using lipid peroxidation (LPO) and 7-ethoxyresorufin O-deethylase (EROD) assays. Docking analysis with Human NAD[P]H-Quinone oxidoreductase 1 (NQO1) was also performed to gather thorough information about these compounds that have antioxidant activities. Moreover, their molecular descriptors and ADME properties were calculated using the SwissADME online program. As a result, most of our compounds possessed better affinity and created ample interactions with NQO1. The most potent compound **5j** had LP inhibition value of 3.73 nmol/mg/min. Other compounds exhibited moderate activity on LP levels comparing to standard butylated hydroxy toluene (**BHT)**. However, the inhibitory effect on EROD activity was not significant.

## 1. Introduction

Antioxidant defense mechanisms, including enzymatic and nonenzymatic systems, play a key role in cellular physiology and survival maintenance by preventing oxidative stress-related cellular damage [1,2]. The imbalance between oxygen-derived free radicals and antioxidant defense systems can lead to this damage by disrupting these antioxidant mechanisms. Eventual antioxidant capacity deficiency can be treated with novel compounds with antioxidant activity and free radical scavenging properties. Lipid peroxidation (LPO), which is one of the most commonly used assays to analyze in vitro antioxidant activity of a new compound, is an oxidative process mediated by reactive oxygen species (ROS) that results in damage to cellular membranes and other lipid-containing structures [3]. ROS can be generated by CYP450 enzymes while they are catalyzing their endogenous and exogenous substrates, and in the end, ROS would lead to the generation of potentially carcinogenic and mutagenic lipid peroxidation (LPO) end products. Among CYP450 enzyme family, CYP1A1 is of great importance in NADPH-dependent LPO. Therefore, probing the effects of antioxidant drug candidates on the catalytic activity of CYP450 enzymes and LPO levels is crucial. [4].

In NADPH-dependent LPO, NAD(P)H:quinone acceptor oxidoreductases (NQO’s) are flavoenzymes that effectively catalyze the reduction of quinone derivatives to hydroquinones [5]. The quinones are electrophilic molecules that can alkylate certain biopolymers such as proteins and DNA in cells. Reduction of quinones with one electron generates the semiquinone radicals and ROS that can induce cellular damage. NQO1, a major member of the NAD(P)H:quinone acceptor oxidoreductases family, contains flavin adenine dinucleotide (FAD) for its stability and proper function and is also highly inducible under oxidative stress. This enzyme can assist the protection of antioxidant substances such as ubiquinone, α-tocopherol quinone, PARP (Poly (ADP-ribose) polymerase) as well as its macromolecular binding functions and may contribute to the cellular protective response. Considering this knowledge about NQO1, a docking analysis has been conducted to identify whether our compounds interact with this enzyme in a similar manner to the literature [6].

Pyrroles and benzimidazole ring systems exhibit various biological activities including antioxidant, antibacterial, and anticancer properties [7–12]. The effects of the benzimidazole ring on the CYP system have been known for a long time. The ability of imidazole derivatives to inhibit some CYP activities has been studied. These studies have shown that the ability of compounds to interact with CYPs is largely dependent on their lipophilic and electronic characters [13–16]. In our previous papers, we applied the strategy of using particular benzimidazole conjugates as antioxidant agents [1,17,18]. In light of data obtained from our previous studies, we still explore the potential new benzimidazole derivatives with strong antioxidant activities. For instance, we have synthesized and characterized some new 6-fluoro-5-substituted-benzimidazole compounds bearing an indole ring at the second position of the benzimidazole ring and then evaluated their antioxidant capacities in vitro. At the 10^–3^ M concentrations, almost all the synthesized compounds exhibited remarkable superoxide anion formation inhibitory effects compared to that of superoxide dismutase [19]. In this study, we have aimed to conjugate pyrrole fragments instead of an indole ring to the benzimidazole from the second position to evaluate their antioxidant activities and structure-activity relationships. Herein, some new 5-(alkylsulfonyl)-1-substituted-2-(1H-pyrrol-2-yl)-1H-benzo[d]imidazole derivatives (**5a–n**) were designed, synthesized, characterized, and were analyzed in silico and in vitro. Their free radical scavenging properties were then probed in vitro by employing lipid peroxidation (LPO) and 7-ethoxyresorufin O-deethylase (EROD) assays to verify their potential antioxidant activities and structure-activity relationship.

## 2. Materials and methods

### 2.1. Chemistry

**Figure 1:**
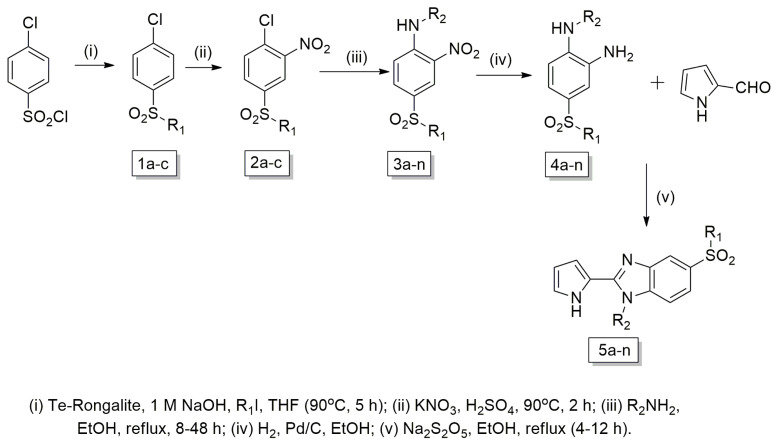
Synthesis procedure of novel pyrrole-benzimidazole derivatives **5a–n**.

Büchi SMP-20 (Büchi Labortechnik, Flawil, Switzerland) and Electrothermal 9100 capillary melting point apparatus (Electrothermal, Essex, UK) were used for determination and uncorrection of the melting points. Varian Mercury-400 FT-NMR spectrometer (Varian Inc., Palo Alto, CA, USA) was used for recording the ^1^H NMR and ^13^C NMR spectra, and LC-MS spectrometer (Waters Corporation, Milford, MA, USA) was used to record the Mass spectra based on ESI(+). LECO 932 CHNS (Leco - 932, St. Joseph, MI, USA) instrument was used for elemental analysis. For column chromatography (cc), silica gel 60 (40–63 mm particle size) was used. 4-Chlorobenzenesulfonylchloride, 1-chloro-4-(methylsulfonyl)benzene (**1a**), and pyrrole-2-carboxaldehyde are commercially available and purchased from Sigma-Aldrich company. Synthesis of new compounds **5a–n** outlined in Figure 1. 4-chloro-benzenesulfonyl chloride was used as starting material (**1**). Compounds **1b, 1c**, and **2a–c** were prepared according to the previous publications [20–24].

#### 2.1.1. General procedure for synthesis of 3a-n

To the solution of 4-(alkylsulfonyl)-1-chloro-2-nitrobenzene (**2a–c**) (5 mmol) in ethanol (5 mL), amine derivative (15 mmol) was added and heated under reflux, until the starting material was consumed (determined by TLC, 8–48 h). Upon cooling the mixture, water was added. The resultant yellow residue was crystallized from ethanol or purified by cc by using a mixture of hexane and ethyl acetate in varying concentrations as eluent [20].

#### 2.1.2. General procedure for synthesis of 4a–n

Compounds **3a–n** (3.5 mmol) in EtOH (75 mL) reduced by hydrogenation using 40 psi of H_2_ and 10% Pd/C (40 mg) until cessation of H_2_ uptake to obtain the catalyst before filtering off on a bed of celite and washing with EtOH; and concentrating the filtrate in vacuo [22]. The crude amine was used without purification [23].

#### 2.1.3. General procedure for synthesis of 5a–n

A mixture of the appropriate *o*-phenylenediamine (**4a–n**, 1 mmol), pyrrole-2-carboxaldehyde (1 mmol) and Na_2_S_2_O_5_ (40%) (2 mL) in EtOH (4 mL), was refluxed until the starting material was consumed (determined by TLC, 4–12 h). The reaction mixture was poured into water, and the precipitate was filtered and washed with water. The residue was purified by cc to give the final product (**5a–n**) (Figure 1) [19,23].

##### 2.1.3.1. 1-Methyl-5-(methylsulfonyl)-2-(1*H*-pyrrol-2-yl)-1*H*-benzo[*d*]imidazole (5a)

Compound **5a** was prepared according to general methods starting from *N*^1^-(methyl)-4-(methylsulfonyl)benzene-1,2-diamine (1.81 mmol, 0.363 g) and pyrrol-3-carboxaldehyde (1.81 mmol, 0.172 g). The residue was purified by cc using the mixture of chloroform - ethyl acetate - hexane (2:2:1) as eluent to give a white solid, m.p. 259 °C (0.045 g, 10% yield). **^1^H NMR (400 MHz, DMSO - *d6*): δ ppm **3.23 (s, 3H), 4.02 (s, 3H), 6.30–6.32 (m, 1H), 6.94 (t, 1H), 7.07 (d, *J* = 1.2 Hz, 1H), 7.76 (dd, *J* = 1.6 Hz, *J* = 8.4 Hz, 1H), 7.84 (d, *J* = 8.8 Hz, 1H), 8.07 (d, *J* = 1.6 Hz, 1H), 11.99 (s, 1H). **^13^C NMR (DMSO - *d6*) δ ppm **31.9, 44.2, 109.7, 110.7, 117.0, 120.0, 120.3, 122.5, 134.2, 139.5, 141.8, 149.9. **MS (ESI +) m/z:** 276. **Anal. calcd. for C_13_H_13_N_3_O_2_S-0.15 H_2_O:** C, 56.15; H, 4.82; N, 15.11; S, 11.53; Found: C, 56.02; H, 4.72; N, 15.10; S, 11.48.

##### 2.1.3.2. 1-Ethyl-5-(methylsulfonyl)-2-(1*H*-pyrrol-2-yl)-1*H*-benzo[*d*]imidazole (5b)

Compound **5b** was prepared according to general methods starting from *N*^1^-(ethyl)-4-(methylsulfonyl)benzene-1,2-diamine (1.03 mmol, 0.222 g) and pyrrol-3-carboxaldehyde (1.03 mmol, 0.097 g). The residue was purified by cc using the mixture of chloroform - ethyl acetate - hexane (2:2:1) as eluent to give a white solid, m.p. 204 °C (0.171 g, 57% yield). **^1^H NMR (400 MHz, DMSO - *d6*): δ ppm **1.40 (t, 3H), 3.23 (s, 3H), 4.54 (q, 2H), 6.31–6.33 (m, 1H), 6.85–6.87 (m, 1H), 7.06–7.07 (m, 1H), 7.77 (dd, *J* = 1.6 Hz, *J* = 8.4 Hz, 1H), 7.86 (d, *J* = 8.4 Hz, 1H), 8.09 (d, *J* = 1.6 Hz, 1H), 11.95 (s, 1H). **^13^C NMR (DMSO - *d6*) δ ppm** 14.5, 44.2, 48.6, 109.9, 110.6, 110.7, 120.0, 120.2, 122.5, 134.4, 138.6, 142.0, 149.0. **MS (ESI +) m/z:** 290. **Anal. calcd. for C_14_H_15_N_3_O_2_S - 0.3 H_2_O:** C, 57.04; H, 5.33; N, 14.25; S, 10.87; Found: C, 56.93; H, 5.35; N, 14.35; S, 11.02.

##### 2.1.3.3. 1-propyl-5-(methylsulfonyl)-2-(1*H*-pyrrol-2-yl)-1*H*-benzo[*d*]imidazole (5c)

Compound **5c** was prepared according to general methods starting from *N*^1^-(propyl)-4-(methylsulfonyl)benzene-1,2-diamine (0.76 mmol, 0.174 g) and pyrrol-3-carboxaldehyde (0.76 mmol, 0.073 g). The residue was purified by cc using the mixture of chloroform - ethyl acetate - hexane (1:2:1) as eluent to give a white solid, m.p. 189 °C (0.053 g, 23% yield). **^1^H NMR (400 MHz, DMSO - *d6*): δ ppm **0.94 (t, 3H), 1.82 (m, 2H), 3.24 (s, 3H), 4.46 (t, 2H), 6.30–6.32 (m, 1H), 6.82 (s, 1H), 7.06 (d, *J* = 0.8 Hz, 1H), 7.76 (dd, *J* = 1.6 Hz, *J* = 8.2 Hz, 1H), 7.87 (d, *J* = 8.8 Hz, 1H), 8.08 (d, *J* = 1.2 Hz, 1H), 11.95 (s, 1H). **^13^C NMR (DMSO - *d6*) δ** 10.8, 22.3, 44.2, 45.4, 109.9, 110.6, 110.9, 117.1, 120.1, 120.1, 122.4, 134.3, 139.1, 141.8, 149.2. **MS (ESI +) m/z:** 304. **Anal. calcd. for C_15_H_17_N_3_O_2_S - 0.2 H_2_O:** C, 58.68; H, 5.71; N, 13.68; S, 10.44; Found: C, 58.36; H, 5.67; N, 13.69; S, 10.41.

##### 2.1.3.4. 1-Butyl-5-(methylsulfonyl-2-(1*H*-pyrrol-2-yl)-1*H*-benzo[*d*]imidazole (5d)

Compound **5d** was prepared according to general methods starting from *N*^1^-(butyl)-4-(methylsulfonyl)benzene-1,2-diamine (0.70 mmol, 0.171 g) and pyrrol-3-carboxaldehyde (0.70 mmol, 0.067 g). The residue was purified by cc using the mixture of chloroform - ethyl acetate - hexane (2:3:3) as eluent to give a white solid, m.p. 151 °C (0.098 g, 44% yield). **^1^H NMR (400 MHz, DMSO - *d6*): δ ppm **0.87 (t, 3H), 1.34 (m, 2H), 1.74 (m, 2H), 3.21 (s, 3H), 4.47 (t, 2H), 6.27–6.29 (m, 1H), 6.80 (s, 1H), 7.03 (d, *J* = 1.2 Hz, 1H), 7.74 (dd, *J* = 1.6 Hz, *J* = 8.8 Hz, 1H), 7.83 (d, *J* = 8.8 Hz, 1H), 8.05 (d, *J* = 1.6 Hz, 1H), 11.92 (s, 1H). **^13^C NMR (DMSO - *d6*) δ** 13.5, 19.3, 31.0, 43.9, 44.1, 109.8, 110.6, 110.8, 117.0, 120.0, 120.1, 122.3, 134.2, 139.0, 141.7, 149.1. **MS (ESI +) m/z:** 318. **Anal. calcd. for C_16_H_19_N_3_O_2_S:** C, 60.20; H, 6.06; N, 13.16; S, 10.04; Found: C, 60.18; H, 6.09; N, 13.29; S, 10.15.

##### 2.1.3.5. 1-Cyclohexyl-5-(methylsulfonyl)-2-(1*H*-pyrrol-2-yl)-1*H*-benzo[*d*]imidazole (5e)

Compound **5e** was prepared according to general methods starting from *N*^1^-(cyclohexyl)-4-(methylsulfonyl)benzene-1,2-diamine (1.00 mmol, 0.27 g) and pyrrol-3-carboxaldehyde (1.00 mmol, 0.096 g). The residue was purified by cc using the mixture of chloroform - ethyl acetate - hexane (1:1.5:0.5) as eluent to give a white solid, m.p. 252 °C (0.138 g, 40% yield). **^1^H NMR (400 MHz, DMSO - *d6*): δ ppm **1.42–1.44 (m, 3H), 1.69 (s, 1H), 1.88–1.96 (m, 4H), 2.26–2.34 (m, 2H), 3.21 (S, 3H), 4.74–4.80 (m, 1H), 6.29 (d, *J* = 1.6 Hz, 1H), 6.60 (t, 1H), 7.04 (s, 1H), 7.68 (dd, *J* = 1.6 Hz, *J* = 8.6 Hz, 1H), 8.05 (s, 1H), 8.07 (d, *J* = 1.6 Hz, 1H), 11.88 (s, 1H). **^13^C NMR (DMSO - *d6*) δ ppm **24.3, 25.4, 30.3, 44.1, 56.6, 109.5, 110.7, 113.4, 117.6, 119.7, 119.8, 122.0, 134.0, 136.9, 142.7, 149.6. **MS (ESI +) m/z:** 344. **Anal. calcd. for C_18_H_21_N_3_O_2_S - 0,3H_2_O:** C, 61.97; H, 6.24; N, 12.04; S, 9.19; Found: C, 61.96; H, 6.15; N, 12.13; S, 9.23.

##### 2.1.3.6. 1-Benzyl-5-(methylsulfonyl)-2-(1*H*-pyrrol-2-yl)-1*H*-benzo[*d*]imidazole (5f)

Compound **5f** was prepared according to general methods starting from *N*^1^-(benzyl)-4-(methylsulfonyl)benzene-1,2-diamine (0.70 mmol, 0.188 g) and pyrrol-3-carboxaldehyde (0.70 mmol, 0.067 g). The residue was purified by cc using the mixture of chloroform - ethyl acetate - hexane (2:3:3) as eluent to give a white solid, m.p. 172 °C (0.096 g, 39% yield). **^1^H NMR (400 MHz, DMSO - *d6*): δ ppm **3.24 (s, 3H), 5.81 (s, 2H), 6.19 (s, 1H), 6.59 (t, 1H), 7.03 (s, 1H), 7.08 (d, *J* = 7.2 Hz, 2H), 7.27.35 (m, 3H), 7.59–7.76 (m, 2H), 8.14 (s, 1H), 12.01 (s, 1H). **^13^C NMR (DMSO - *d6*) δ ppm** 44.1, 47.3, 109.8, 110.9, 111.1, 117.2, 119.8, 120.5, 122.6, 125.9, 127.4, 128.8, 134.7, 136.3, 139.4, 142.0, 149.7. **MS (ESI +) m/z:** 352. **Anal. calcd. for C_19_H_17_N_3_O_2_S - 0.4 H_2_O:** C, 63.63; H, 5.00; N, 11.71; S, 8.94; Found: C, 63.24; H, 4.86; N, 11.64; S, 8.87.

##### 2.1.3.7. 1-(4-Fluorobenzyl)-5-(methylsulfonyl)-2-(1*H*-pyrrol-2-yl)-1*H*-benzo[*d*]imidazole (5g)

Compound **5g** was prepared according to general methods starting from *N*^1^-(4-fluorobenzyl)-4-(methylsulfonyl)benzene-1,2-diamine (0.94 mmol, 0.277 g) and pyrrol-3-carboxaldehyde (0.94 mmol, 0.089 g). The residue was purified by cc using the mixture of chloroform - ethyl acetate - hexane (2:3:3) as eluent to give a white solid, m.p. 162 °C (0.051 g, 15% yield). **^1^H NMR (400 MHz, DMSO - *d6*): δ ppm **3.22 (s, 3H), 5.77 (s, 2H), 6.18 (s, 1H), 6.59 (s, 1H), 7.01 (s, 1H), 7.08–7.17 (m, 4H), 7.72–7.77 (m, 2H), 8.11 (1H), 11.99 (s, 1H). **^13^C NMR (DMSO - *d6*) δ ppm **44.1, 46.7, 109.8, 110.9, 110.1, 115.7 (d, *J =* 21.4 Hz), 117.3, 119.6, 122.7, 128.07 (d, *J* = 7.6 Hz), 132.5 (d, *J* = 3.1 Hz), 134.8, 139.2, 142.6, 161.3 (d, *J* = 242.3 Hz). **MS (ESI+) m/z:** 370. **Anal. calcd. for C_19_H_16_FN_3_O_2_S - 0.45 H_2_O:** C, 60.44; H, 4.51; N, 11.13; S, 8.49; Found: C, 60.18; H, 4.36; N, 11.06; S, 8.60.

##### 2.1.3.8. 1-(4-Chlorobenzyl)-5-(methylsulfonyl)-2-(1*H*-pyrrol-2-yl)-1*H*-benzo[*d*]imidazole (5h)

Compound **5h** was prepared according to general methods starting from *N*^1^-(4-chlorobenzyl)-4-(methylsulfonyl)benzene-1,2-diamine (0.78 mmol, 0.243 g) and pyrrol-3-carboxaldehyde (0.78 mmol, 0.075 g). The residue was purified by cc using the mixture of chloroform - ethyl acetate - hexane (2:3:3) as eluent to give a white solid, m.p. 193 °C (0.113 g, 37% yield). **^1^H NMR (400 MHz, DMSO - *d6*): δ ppm** 3.25 (s, 3H), 5.81 (s, 2H), 6.19 - 6.21 (m, 1H), 6.57–6.59 (m, 1H), 7.03–7.04 (m, 1H), 7.10 (d, *J* = 8.0 Hz, 2H), 7.38–7.42 (m, 2H), 7.76–7.77 (m, 2H), 8.14 (d, *J* = 0.8 Hz, 1H), 12.02 (s, 1H). **^13^C NMR (DMSO - *d6*) δ ppm **44.1, 46.7, 109.8, 110.9, 111.1, 117.3, 119.7, 120.6, 122.7, 127.8, 128.8, 132.0, 134.8, 135.4, 139.2, 142.0, 149.6. **MS (ESI +) m/z:** 386. **Anal. calcd. for C_19_H_16_ClN_3_O_2_S - 0.1 H_2_O:** C, 58.86; H, 4.21; N, 10.83; S, 8.27; Found: C, 58.74; H, 4.19; N, 10.85; S, 8.26.

##### 2.1.3.9. 1-Propyl-5-(ethylsulfonyl)-2-(1*H*-pyrrol-2-yl)-1*H*-benzo[*d*]imidazole (5i)

Compound **5i** was prepared according to general methods starting from *N*^1^-(propyl)-4-(ethylsulfonyl)benzene-1,2-diamine (0.88 mmol, 0.215 g) and pyrrol-3-carboxaldehyde (0.88 mmol, 0.085 g). The residue was purified by cc using the mixture of chloroform - ethyl acetate - hexane (2:1:1) as eluent to give a white solid, m.p. 146 °C (0.046 g, 16% yield). **^1^H NMR (400 MHz, DMSO - *d6*): δ ppm **0.94 (t, 3H), 1.12 (t, 3H), 1.80–1.85 (m, 2H), 3.31 (q, 2H), 4.46 (t, 2H), 6.31 (m, 1H), 6.82 (s, 1H), 7.06 (d, *J* = 0.8 Hz, 1H), 7.71 (dd, *J* = 1.6 Hz, *J* = 9.0 Hz, 1H), 7.88 (d, *J* = 8.8 Hz, 1H), 8.03 (d, *J* = 1.6 Hz, 1H), 11.95 (s, 1H). **^13^C NMR (DMSO - *d6*) δ ppm **7.3, 10.8, 22.3, 45.5, 49.7, 109.9, 110.7, 110.9, 117.9, 120.1, 120.9, 122.4, 131.8, 139.3, 141.9, 149.2. **MS (ESI +) m/z:** 318. **Anal. calcd. for C_16_H_19_N_3_O_2_S:** C, 60.54; H, 6.03; N, 13.23; S, 10.10; Found: C, 60.68; H, 6.23; N, 13.14; S, 10.07.

##### 2.1.3.10. 1-Benzyl-5-(ethylsulfonyl)-2-(1*H*-pyrrol-2-yl)-1*H*-benzo[*d*]imidazole (5j)

Compound **5j** was prepared according to general methods starting from *N*^1^-(benzyl)-4-(ethylsulfonyl)benzene-1,2-diamine (0.95 mmol, 0.277 g) and pyrrol-3-carboxaldehyde (0.95 mmol, 0.091 g). The residue was purified by cc using the mixture of chloroform - ethyl acetate - hexane (2:1:1) as eluent to give a white solid, m.p. 183 °C (0.130 g, 38% yield). **^1^H NMR (400 MHz, DMSO - *d6*): δ ppm **1.10 (t, 3H), 3.29 (q, 2H), 5.78 (s, 2H), 6.15–6.18 (m, 1H), 6.56 (s, 1H), 7.01 (s, 1H), 7.06 (d, *J* = 8.4 Hz, 2H), 7.24 (d, *J* = 8.4 Hz, 1H), 7.30 (t, 2H), 7.69 (dd, *J* = 1.2 Hz, *J* = 8. Hz, 1H), 7.75 (d, *J* = 8.0 Hz, 1H), 8.07 (d, *J* = 1.2 Hz, 1H), 12.00 (s, 1H). **^13^C NMR (DMSO - *d6*) δ ppm **7.3, 47.3, 49.6, 109.8, 111.0, 111.1, 118.1, 119.8, 121.3, 122.7, 125.9, 127.5, 128.9, 132.2, 136.4, 139.5, 142.0, 149.7. **MS (ESI +) m/z:** 366. **Anal. calcd. for C_20_H_19_N_3_O_2_S:** C, 65.73; H, 5.24; N, 11.50; S, 8.77; Found: C, 65.81; H, 5.39; N, 11.26; S, 8.68.

##### 2.1.3.11. 1-(4-Fluorobenzyl)-5-(ethylsulfonyl)-2-(1*H*-pyrrol-2-yl)-1*H*-benzo[*d*]imidazole (5k)

Compound **5k** was prepared according to general methods starting from *N*^1^-(p-fluorobenzyl)-4-(ethylsulfonyl)benzene-1,2-diamine (0.62 mmol, 0.193 g) and pyrrol-3-carboxaldehyde (0.62 mmol, 0.060 g). The residue was purified by cc using the mixture of chloroform - ethyl acetate - hexane (2:1:1) as eluent to give a white solid, m.p. 164 °C (0.058 g, 24% yield)**. ^1^H NMR (400 MHz, DMSO - *d6*): δ ppm** 1.12 (t, 3H), 3.31 (q, 2H), 5.79 (s, 2H), 6.20 (q, 1H), 6.61 (s, 1H), 7.03–7.04 (m, 1H), 7.11–7.19 (m, 4H), 7.70 (dd, *J* = 1,6 Hz, *J* = 8.4 Hz, 1H), 7.79 (d, *J* = 8.8 Hz, 1H), 8.08 (d, *J* = 1.2 Hz, 1H), 12.00 (s, 1H). **MS (ESI +) m/z:** 384. **Anal. calcd. for C_20_H_18_FN_3_O_2_S - 0.1H_2_O:** C, 62.35; H, 4.76; N, 10.90; S, 8.32; Found: C, 62.16; H, 4.98; N, 10.77; S, 8.20.

##### 2.1.3.12. 1-(3,4-Difluorobenzyl)-5-(ethylsulfonyl)-2-(1*H*-pyrrol-2-yl)-1*H*-benzo[*d*]imidazole (5l)

Compound **5l** was prepared according to general methods starting from *N*^1^-(3,4-difluorobenzyl)-4-(ethylsulfonyl)benzene-1,2-diamine (0.77 mmol, 0.254 g) and pyrrol-3-carboxaldehyde (0.77 mmol, 0.074 g). The residue was purified by cc using the mixture of chloroform - ethyl acetate - hexane (2:2:1) as eluent to give a white solid, m.p. 182 °C (0.112 g, 36% yield). **^1^H NMR (400 MHz, DMSO - *d6*): δ ppm **1.13 (t, 3H), 3.32 (q, 2H), 5.81 (s, 2H), 6.22 (q, 1H), 6.62 (s, 1H), 6.82 (d, *J* = 8.0 Hz, 1H), 7.06 (d, *J* = 0.8 Hz, 1H), 7.26–7.42 (m, 2H), 7.72 (dd, *J* = 1.6 Hz, *J* = 8.4 Hz, 1H), 7.81 (d, *J* = 8.4 Hz, 1H), 8.10 (d, *J* = 1.6 Hz, 1H), 12.03 (s, 1H). **^13^C NMR (DMSO - *d6*) δ ppm **7.2, 46.3, 49.6, 109.8, 110.8, 111.0, 115.4, 115.6, 117.9, 118.0, 118.1, 119.6, 121.3, 122.7, 132.4, 139.2, 142.05, 149.5, 149.7. **MS (ESI+) m/z:** 402. **Anal. calcd. for C_20_H_17_F_2_N_3_O_2_S:** C, 59.83; H, 4.26; N, 10.46; S, 7.98; Found: C, 59.60; H, 4.28; N, 10.42; S, 8.02.

##### 2.1.3.13. 1-(3,4-Dichlorobenzyl)-5-(ethylsulfonyl)-2-(1*H*-pyrrol-2-yl)-1*H*-benzo[*d*]imidazole (5m)

Compound **5m** was prepared according to general methods starting from *N*^1^-(3,4-dichlorobenzyl)-4-(ethylsulfonyl)benzene-1,2-diamine (0.62 mmol, 0.225 g) and pyrrol-3-carboxaldehyde (0.62 mmol, 0.059 g). The residue was purified by cc using the mixture of chloroform - ethyl acetate - hexane (2:2:1) as eluent to give a white solid, m.p. 191 °C (0.062 g, 23% yield). **^1^H NMR (400 MHz, DMSO - *d6*): δ ppm **1.12 (t, 3H), 3.32 (q, 2H), 5.83 (s, 2H), 6.21 (q, 1H), 6.60 (s, 1H), 6.93 (dd, *J* = 1.6 Hz, *J* = 8.6 Hz, 1H), 7.05 (s, 1H), 7.47 (d, *J* = 1.6 Hz, 1H), 7.57 (d, *J* = 8.8 Hz, 1H), 7.7 (dd, *J* = 1.6 Hz, *J* = 8.8 Hz, 1H), 7.80 (d, *J* = 8.0 Hz, 1H), 8.09 (d, *J* = 1.2 Hz, 1H), 12.03 (s, 1H). **^13^C NMR (DMSO - *d6*) δ ppm **7.3, 46.3, 49.6, 109.9, 110.9, 111.1, 118.2, 119.6, 121.5, 122.9, 126.1, 128.3, 130.1, 131.1, 131.4, 132.5, 137.6, 139.3, 142.1, 149.6. **MS (ESI+) m/z:** 435. **Anal. calcd. for C_20_H_17_Cl_2_N_3_O_2_S:** C, 55.30; H, 3.94; N, 9.67; S, 7.38; Found: C, 55.33; H, 3.94; N, 9.78; S, 7.39.

##### 2.1.3.14. 1-(3,4-Dichlorobenzyl)-5-(propylsulfonyl)-2-(1*H*-pyrrol-2-yl)-1*H*-benzo[*d*]imidazole (5n)

Compound **5n** was prepared according to general methods starting from *N*^1^-(3,4-dichlorobenzyl)-4-(propylsulfonyl)benzene-1,2-diamine (0.61 mmol, 0.228 g) and pyrrol-3-carboxaldehyde (0.61 mmol, 0.058 g). The residue was purified by cc using the mixture of chloroform - ethyl acetate - hexane (2:3:3) as eluent to give a white solid, m.p. 196 °C (0.075 g, 27% yield). **^1^H NMR (400 MHz, DMSO - d6): δ ppm **0.91 (t, 3H), 1.55 – 1.61 (m, 2H), 3.30 (t, 2H), 5.82 (s, 2H), 6.20–6.22 (m, 1H), 6.59 (s, 1H), 6.92 (dd, *J* = 2.0 Hz, *J* = 9.0 Hz, 1H), 7.04–7.05 (m, 1H), 7.46 (d, *J* = 2.0 Hz, 1H), 7.57 (d, *J* = 8.0 Hz, 1H), 7.71 (dd, *J* = 2.0 Hz, *J* = 9.0 Hz, 1H), 7.80 (d, *J* = 8.8 Hz, 1H), 8.08 (d, *J* = 1.6 Hz, 1H), 12.03 (s, 1H). **^13^C NMR (DMSO - d6) δ** 12.5, 16.3, 46.3, 56.7, 109.9, 110.8, 111.1, 118.0, 119.6, 121.3, 122.8, 126.1, 128.3, 130.1, 131.1, 131.4, 133.1, 137.6, 139.3, 142.0, 149.5. **MS (ESI+) m/z:** 449. **Anal. calcd. for C_21_H_19_Cl_2_N_3_O_2_S:** C, 56.25; H, 4.27; N, 9.37; S, 7.15; Found: C, 55.96; H, 4.25; N, 9.46; S, 7.19.

### 2.2. In vitro antioxidant activity

#### 2.2.1. Treatment of animals

Albino male Wistar rats with 200–225 g were used in the present study. They were individually housed in standard cages with free access to tap water and standard rat chow ad libitum and maintained at room temperature of 22–25 °C, a 12 h light-dark cycle, and 60% relative humidity. Rats were deprived of feed for 24 h before decapitation under anaesthesia. The liver tissues were dissected quickly, rinsed thoroughly with deionized water, weighed, and then immediately stored at −80 °C to minimize any potential changes before processing. All housing and experimental procedures were approved by Ankara University Animal Ethics Committee.

#### 2.2.2. Isolation of rat liver microsomes

Rat liver tissues were homogenized in cold 1.15% KCI (w/v) with a homogenizer on ice at 250 x g. The homogenates were first centrifuged at 11,000 x g for 25 min at 4 °C and the resulting supernatants were collected and further ultracentrifuged at 108,000 x g for 60 min at 4 °C. The latter pellets were suspended with 20% glycerol and maintained at −80 °C until further analysis. Total microsomal protein concentrations were quantified by the protocol of Lowry et al. [25], using bovine serum albumin as a standard.

#### 2.2.3. Lipid peroxidation (LPO) assay

Lipid peroxidation (LPO) level was determined based on the protocols reported by Wills [26,27], and Bishayee and Balasubramanian [28], with some modifications described previously [29]. The assay employs the measurement of thiobarbituric acid reactive substances (TBARS) that yields a pink color and can be measured spectrophotometrically at 532 nm, resulting from the reaction between lipid peroxidation products, mainly malondialdehyde (MDA), and thiobarbituric acid (TBA) under acidic conditions and high temperature. The final reaction mixture (1 mL) in the test tube consists of 0.2 mg microsomal protein, 10^–3^ M test compound, 62.5 mM potassium phosphate buffer (pH: 7.4), 90 mM KCl, 0.2 mM Fe^2+^, in which cofactor (NADPH - generating system containing of 2.5 mM MgCI_2_, 0.25 mM NADP^+^, 2.5 mM glucose-6-phosphate, 14.2 mM potassium phosphate buffer (pH 7.8), and 1.0 U glucose-6-phosphate dehydrogenase) was added to initiate the reaction. Next, the mixture was incubated at 37 °C in a shaking water bath. After 30 min of incubation, the reaction was terminated by the addition of 500 μL of 25 % (w/v) trichloroacetic acid (TCA) and the mixture was then centrifuged at 7000 x g for 20 min to remove denatured proteins. One milliliter of the supernatant was mixed with 0.5 mL of TBA and then heated for 20 min in a boiling water bath. The absorbance of TBARS was measured at 532 nm against the blank, which contains all reagents without microsomal proteins. The results were then expressed as nmol TBARS/mg of protein. In this protocol, while dimethyl sulfoxide (DMSO), in which the synthesized compounds were dissolved, was employed as the control, butylated hydroxyl toluene (BHT) was used as a standard.

#### 2.2.4. 7-Ethoxyresorufin O-deethylase (EROD) assay

7-Ethoxyresorufin O-deethylase (EROD) activity was assayed by the protocol described by Burke et al. [30]. EROD activity is measured by following the CYP1A1-mediated deethylation of the substrate 7-ethoxyresorufin to form the product resorufin that can be monitored fluorometrically [30]. The final reaction mixture (1 mL) in the test tube consists of 0.2 mg microsomal protein, 10^–3^ M test compound, 1.0 mM 7-ethoxyresorufin as a substrate, 12 mM albumin, 100 mM Tris – HCl buffer (pH 7.8), in which cofactor (NADPH - generating system consisting of 0.25 mM NADP^+^, 2.5 mM MgCI_2_, 2.5 mM glucose-6-phosphate, 14.2 mM potassium phosphate buffer (pH 7.8), and 1.0 U glucose-6-phosphate dehydrogenase) was added to initiate the reaction. Next, the mixture was incubated at 37 °C for 5 min in a shaking water bath. After incubation, the reaction was terminated by the addition of 3 mL ice cold methanol and the mixture was then centrifuged at 7 000 x g for 20 min to remove denatured proteins. Following the centrifugation, the fluorescence intensity of the supernatant (3 mL) was read at the excitation/emission wavelengths of 538 nm/587 nm. While caffeine was employed as a standard, the control used in this protocol was dimethyl sulfoxide (DMSO) in which the synthesized compounds were dissolved.

#### 2.2.5. Docking method and ADME property calculation

Human NAD[P]H-Quinone oxidoreductase 1 (PDB ID : 1dxo, resolution: 2.5 Å) file was obtained from the RCSB Protein Data Bank website [31]. AutoDockTools v.1.5.6 was used for deleting water molecules and defining the grid box [32]. Following this process, polar hydrogens and Gasteiger charges were added, and the grid was also prepared using the same software. Assigned grid’s center were X = –2.718, Y = 16.674, Z = 5.139 and dimensions were X = 40, Y = 40, Z = 40. Spacing was defined as 0.375 Å. The 2D structures of the compounds were drawn on ChemDraw Ultra 12.0, minimized with MMFF94 and UFF force fields (number of steps: 5000 with steepest descent algorithm and convergence value of 10e-7) and then these files were converted to pdb files using Avogadro software [33]. Subsequently, Gasteiger charges and torsion were added to ligand files with AutoDockTools. Prepared ligands were docked with AutoDock Vina [34]. Finally, 3-D docked poses and interaction diagrams of the ligands were generated and interpreted using Discovery Studio Visualizer Ligand interaction module [35].

The evaluation of physicochemical properties and the prediction of ADME parameters were determined with SwissADME online tools [36]. Besides, using BOILED-Egg representation brain penetration and gastrointestinal absorption of synthesized compounds were assessed [37]. With the aid of the abovementioned calculations, we aimed to gather medicinal chemistry information about our compound set and to suggest further solutions for the improvement of these properties.

#### 2.2.6. Validation of the docking method

This molecular docking method was validated for proving the reliability of the results. For this purpose, DockRMSD online program was used. This program can calculate RMSD value between two docked poses of a ligand and can render the result as text output [38]. For the validation of the docking method, co-ligand duroquinone was extracted from the protein file (pdb id:1dxo) then after adding the polar hydrogens and Gasteiger charges, docked again with the same protein. After this, docked and crystallographic poses of duroquinone were converted to convenient format (.pdbqt to .mol2) using Open Babel GUI [39]. In the end, these two files were submitted as input to DockRMSD online program.

## 3. Results

### 3.1. Chemistry

Synthesis of novel pyrrole-benzimidazoles **5a–n** outlined in Figure 1 begins with 4-chloro-benzenesulfonyl chloride as a starting material. Alkylation of the sulfonyl chlorides with iodoalkanes in the presence of tellurium, rongalite, and 1 M aqueous sodium hydroxide gave alkylsulfonyl derivatives (**1b, 1c**) [18]. This was followed by reaction with conc. H_2_SO_4_ and potassium nitrate to give nitro compounds (**2a–c**) [18]. Aromatic nucleophilic substitution of the chlorine atom of 4-(alkylsulfonyl)-1-chloro-2-nitrobenzene (**2a–c**) with appropriated amines to provide the corresponding N-alkyl-4-(alkylsulfonyl)-2-nitroaniline derivatives **3a–n** in good yields [19,22]. Reduction of nitro group yielded to the crude amines **4a–n** [22] and these amines were used without purification. The resulting compounds **(5a–n)** were obtained by reacting o-phenylenediamines **4a–n** with pyrrole-2-carboxaldehyde in the presence of Na_2_S_2_O_5_ and EtOH [22–24]. Yields were not optimized.

### 3.2. Biological activity

The in vitro effects of these novel pyrrole-benzimidazole derivatives on rat liver microsomal NADPH-dependent lipid peroxidation (LPO) levels and ethoxyresorufin O-deethylase (EROD) activity were monitored [9]. When compared to that observed for standard BHT, all synthesized compounds had moderate LPO inhibitory activity, particularly compounds **5b**, **5d**, and **5i–m** displayed high inhibitory activity on LPO with the inhibition rates of 77%–65%. However, none of these compounds had a marked inhibitory effect on EROD activity.

According to the comparison of substituents and activity results which are given in Table 1, we observe that when R_1_ substituent is ethyl, the activity becomes elevated dramatically. Also, by using our R_1_ ethyl-substituted compounds as a starting point, we can determine that nucleophilic groups such as benzyl at R_2_ also would enhance the activity. Benzyl group needs to maintain electron density, therefore we encounter the decreasing values for compounds that are substituted with deactivating halogens: **5k**, **5l**, **5m**, and **5n**. If we examine **5b**, **5d**, and **5e** closely, R_1_ is methyl and R_2_ is ethyl. For **5d** (R_2_ = butyl) and **5e** (R_2_ = cyclohexyl), the lipid peroxidation inhibition slightly decreases. Moreover, the poor activity of compound **5n** can be explained by having propyl at R_1_ and an electronically sparse aromatic ring at R_2_. **5m** also has this aromatic portion at R_2_ but has ethyl instead of propyl at R_1_. This should possibly explain the relatively poor activity of **5n**. According to the LPO assay; compounds **5b**, **5d**, **5i**, **5j**, **5k**, **5l**, **5m** were elected as candidates. These compounds were compared to standard compound **BHT**. Among them, the most potent compound was **5j**.

### 3.3. Molecular docking and ADME studies

**Figure 2:**
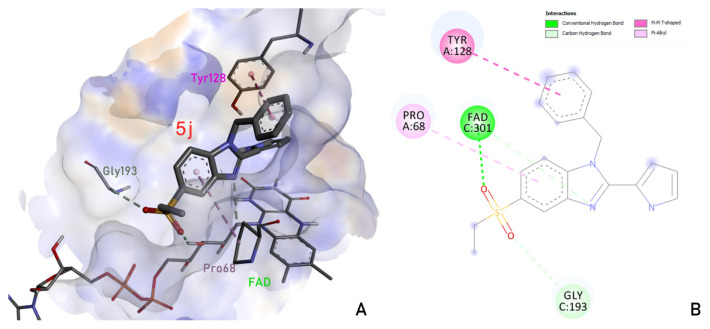
Binding pose (A) and ligand interaction diagram (B) of the most potent compound.

Initially, necessary positioning and interactions have been understood with the aid of a reference study, which mandates that the bound acceptor’s aromatic moieties should become stacked with FAD and maintain certain interactions. Moreover, ligand should offer several interactions with aromatic residues such as Trp105, Phe106, Phe178, Tyr126, and Tyr128 and polar residue His161 [6]. Regarding Figure 2, compound **5j** docked into the binding site while maintaining hydrophobic interactions with Tyr128 through its benzyl substituent and benzimidazole moiety also constitutes Pi-alkyl interaction with Pro68. We can determine that the sulfonyl group acts as an acceptor in H-bond interactions with existing coenzyme FAD and Gly193.

The reliability of this method was proved using the DockRMSD distance calculation program. Out of 1,048,576 possible mappings, an optimal mapping was chosen by the same program. RMSD value for this mapping was calculated as 1.270 Å. For a reliable docking method, this value should be below 2 Å [40]. Fortunately, the RMSD value for these two duroquinone ligands was below this upper limit, therefore our method was proved to be rather versatile and reliable in conducting docking analysis with 1dxo.

The H-bond donor/acceptor count represents the atoms that are capable of interacting with the polar residues in the domains or cavities in the protein besides having an impact on P-glycoprotein transport, permeability, and many other parameters [36,41]. Number of rotatable bonds increases the overall flexibility of the compounds and give them the ability to interact with various enzymes and plasma proteins which have pharmacokinetic functions [36]. Using the Lipinski’s rule of five (RO5) we can predict the oral activity of small molecules. This rule includes the following thresholds: the molecular mass should be below 500 daltons, calculated logP (clogP) must be less than 5.5, hydrogen bond donors should be less than or equal to 5 and hydrogen bond acceptors should be less than or equal to 10. Ligands that fail to conform to at least one of these parameters are flagged as orally undesirable [36,42,43]. Muegge’s filter consists of the limits: 200 ≤ molecular weight ≤ 600, −2 ≤ XLOGP ≤ 5, total polar surface area ≤ 150, the number of rings ≤ 7, the number of carbon > 4, the number of heteroatoms > 1, the number of rotatable bonds ≤ 15, the hydrogen bond acceptors ≤ 10, and the hydrogen bond donors ≤ 5 [44]. Leadlikeness is on the other hand, is a rule-based method that includes 250 ≤ molecular weight ≤ 350, XLOGP ≤ 3.5, number of rotatable bonds ≤ 7 [36, 45].

Moreover, according to Table 2, compounds in our pyrrole-benzimidazole set have exhibited much better docking scores, interactions, and overall drug-likeness. Amongst these derivatives, docking scores for most effective LP inhibitors were found to be relatively lower than BHT and also other compounds. Additionally, the compounds **5g**, **5h**, **5l**, **5m**, and **5n** offered the lowest energy values and **5b**, **5d**, and **5i** passed all of the medicinal chemistry friendliness filters. Since the binding region consists of hydrophobic residues, H-bond acceptor/donor count has lost its relevance. One must note that all these parameters may affect the binding of drug-like compounds to proteins although a certain correlation is yet to be created. We have the knowledge that these ADME properties mainly have an impact on the bioavailability of substances.

**Figure 3:**
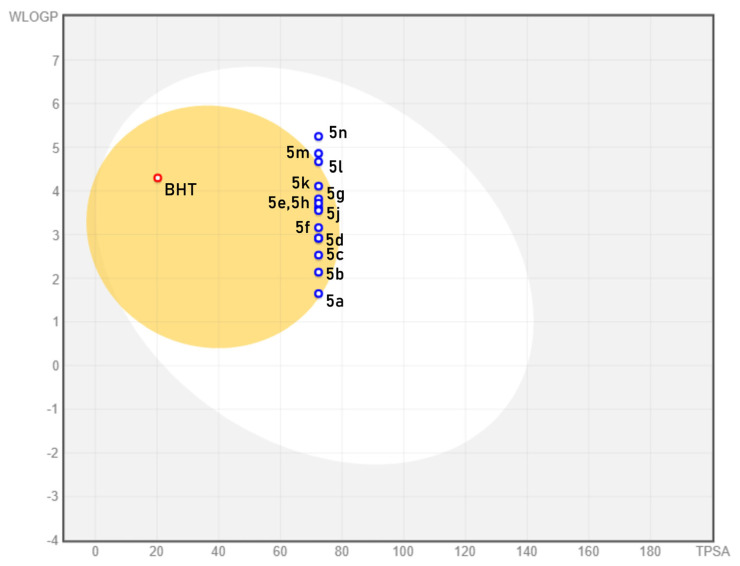
BOILED-Egg representation of pyrrole-benzimidazole derivatives and standard BHT. Compounds are represented as circles in this diagram. Circles that are located in the egg yolk area are predicted to passively permeate through the blood-brain barrier, and the white area covers the compounds which can be passively reabsorbed from the gastrointestinal tract. Red colour defines that the compound is not a substrate for P-glycoprotein while blue colour suggests the opposite. WLOGP and TPSA (topological polar surface area) are computational values that can be calculated in SwissADME.

The BOILED-Egg representation is a useful tool for the interpretation of permeation to the central nervous system (CNS) and gastrointestinal reabsorption. As result, all of the synthesized derivatives were found to be passively absorbable via the gastrointestinal tract. This finding increases the overall bioavailability of our compounds. Hereby we can determine that BHT can effectively pass through the blood-brain barrier (BBB) thus may cause mild CNS adverse effects. Besides, we can witness similar but milder hazards for the compounds except **5l**, **5m**, and **5n**. Among these, **5n** was found to have a much higher WLOGP (Figure 3). This may prevent the permeation of the compound through BBB thus having fewer adverse effects on CNS. These compounds have good absorption through the gastrointestinal tract while possessing a lower chance of permeating through BBB. Fortunately, they also exhibit high antioxidant activity. Overall, these attributes render these three compounds safe and versatile lead-like compounds.

## 4. Discussion

In this study, novel pyrrole-benzimidazole derivatives were designed, synthesized, and characterized and their antioxidant activities were then analyzed through lipid peroxidation and EROD activity. These derivatives exhibited moderate activities relative to BHT. Among all of them, compound **5j** emerged as a potential LPO inhibitor while having no considerable inhibitory effect against EROD activity. This characteristic may increase the chance of it being a potential agent against lipid peroxidation. SAR studies suggested that when R_1_ becomes ethyl and R_2_ is benzyl, the activity greatly enhances. Though when this benzyl group is substituted with the halogen group, activity is decreased.

Results of our studies suggest that pyrrole-benzimidazole derivatives have expressed much higher affinity values and a higher number of interactions in docking analysis, compared to that of standard BHT. Among them, compound **5j** which ensures certain interactions with the enzyme has shown promise as potential lipid peroxidation (LPO) inhibitor. This compound has offered hydrophobic interactions with Tyr128, a steric interaction with Pro68, and H-bond interactions with FAD and Gly193. Pharmacokinetic parameters were also calculated to discuss whether our compounds lack certain requirements for their pharmaceutical development or need certain improvements in this area. Among our derivatives, **5b** and **5d** have passed all the filters although other derivatives failed these requirements. These approaches have helped our team to both discover a novel potent antioxidant agent and to gain a better understanding of certain drug-protein interactions which play a role in this particular activity.

## Figures and Tables

**Table 1:** The inhibition values of synthesized pyrrole-benzimidazole derivatives **5a–n**, standards **BHT** and **Caffeine**, and control **DMSO** against EROD and LPO.

Chem no.	R_1_	R_2_	EROD (pmol/mg/min)	% of control	LPO (nmol/mg/min)	% of control
**5a**	−CH_3_	−CH_3_	44.25 ± 0.79	107	6.95 ± 1.76	43
**5b**	−CH_3_	−C_2_H_5_	49.83 ± 0.09	120	4.83 ± 0.98	30
**5c**	−CH_3_	−C_3_H_7_	44.90 ± 0.67	108	6.98 ± 0.66	43
**5d**	−CH_3_	−C_4_H_9_	44.43 ± 1.29	107	5.61 ± 0.33	35
**5e**	−CH_3_	−cyclohexyl	24.83 ± 1.01	60	16.94 ± 2.75	104
**5f**	−CH_3_	−benzyl	44.17 ± 1.01	106	8.66 ± 1.33	53
**5g**	−CH_3_	−4-fluorobenzyl	44.64 ± 0.41	107	11.08 ± 0.19	68
**5h**	−CH_3_	−4-chlorobenzyl	45.37 ± 0.51	109	10.00 ± 0.99	62
**5i**	−C_2_H_5_	−C_3_H_7_	46.27 ± 1.22	111	5.07 ± 0.49	31
**5j**	−C_2_H_5_	−benzyl	45.79 ± 0.19	110	3.73 ± 0.49	23
**5k**	−C_2_H_5_	−p-fluorobenzyl	44.15 ± 0.75	106	5.37 ± 0.92	33
**5l**	−C_2_H_5_	−3,4-difluorobenzyl	44.63 ± 0.21	107	5.11 ± 0.75	31
**5m**	−C_2_H_5_	−3,4-dichlorobenzyl	44.83 ± 0.72	108	4.70 ± 0.29	29
**5n**	−C_3_H_7_	−3,4-dichlorobenzyl	21.61 ± 2.00	52	15.51 ± 0.78	95
**BHT**			-		5.68 ± 0.22	35
**Caffeine**			6.41 ± 0.36	15	-	-
**DMSO**			41.53 ± 0.99	100	16.25 ± 1.45	100

**Table 2:** AutoDock Vina docking scores and SwissADME results of pyrrole-benzimidazole derivatives and BHT.

No.	Docking score (kcal/mol)	Interacting residues	Count of H-bond acceptors/donors	Number of rotatable bonds	Consensus computational LogP	Lipinski violations	Muegge Filter Violations	Leadlikeness
**5a**	−7.7	Tyr126 – H-bond Tyr126 – Pi-Pi Trp105 – Pi-Pi FAD – H-bond FAD – Pi-alkyl	4/1	2	0.92	0	0	Yes
**5b**	−8.0	Tyr128 – Pi-alkyl Tyr126 – Pi-Pi Trp105 – Pi-Pi His161 – Pi-alkyl FAD – H-bond FAD – Pi-Pi	4/1	3	1.20	0	0	Yes
**5c**	−8.1	Tyr126 – Pi-Pi Trp105 – Pi-Pi Phe178 – Pi-alkyl His161 – Pi-alkyl FAD – H-bond FAD – Pi-Pi FAD – Pi-alkyl	4/1	4	1.51	0	0	Yes
**5d**	−7.7	Gly149 – Carbon H-bond Tyr128 – Pi-Pi Tyr128 – Pi-alkyl Tyr126 – Pi-alkyl Gln66 – H-bond Pro68 – Pi-alkyl FAD – H-bond FAD – Carbon H-bond FAD – Pi-Pi	4/1	5	1.78	0	0	Yes
**5e**	−8.3	Tyr128 – Pi-Pi Tyr128 – Pi-alkyl Gly149 – Carbon H-bond Pro68 – Pi-alkyl FAD – H-bond FAD – Carbon H-bond FAD – Pi-Pi	4/1	3	2.13	0	0	Yes
**5f**	−8.6	Tyr128 – Pi-Pi Pro68 – Pi-alkyl FAD – H-bond FAD – Carbon H-bond FAD – Pi-Pi	4/1	4	2.07	0	0	No; MW>350
**5g**	−9.2	Tyr128 – Pi-Pi Tyr126 -Carbon H-bond Tyr126 – Pi-Pi Tyr126 – Pi-alkyl Phe178 - Pi-alkyl Trp105 - Pi-alkyl Glu123 – H-bond FAD – Pi-Pi FAD – Pi-alkyl	4/1	4	2.56	0	0	No; MW>350
**5h**	−9.0	Tyr128 - Pi-Pi Tyr126 - Carbon H-bond Glu123 – H-bond Pro68 – Pi-alkyl FAD – Pi-Pi	5/1	4	2.37	0	0	No; MW>350
**5i**	−8.1	Tyr126 – Pi-Pi Trp105 – Pi-Pi Phe178 – Pi-alkyl His161 – Pi-alkyl FAD – Pi-Pi FAD – Pi-sigma	4/1	5	1.78	0	0	Yes
**5j**	−8.4	Tyr128 – Pi-Pi Gly193 – Carbon H-bond Pro68 – Pi-alkyl FAD – H-bond FAD - Carbon H-bond	4/1	5	2.35	0	0	No; MW>350
**5k**	−8.9	Tyr128 – Pi-Pi Tyr126 – Pi-donor H-bond Tyr126 – Pi-Pi Glu123 – H-bond Pro68 – Pi-alkyl FAD – Pi-Pi	5/1	5	2.64	0	0	No; MW>350
**5l**	−9.2	Tyr128 – Pi-Pi Tyr126 – Pi-donor H-bond Tyr126 – Pi-Pi Glu123 – H-bond Pro68 – Pi-alkyl FAD – Pi-Pi	6/1	5	2.93	0	0	No; MW>350
**5m**	−9.3	Tyr128 – Pi-Pi Tyr126 – Pi-donor H-bond Tyr126 – Pi-Pi Glu123 – H-bond FAD – Pi-Pi	4/1	5	3.31	0	0	No; MW>350, XLOGP3>3.5
**5n**	−9.4	Tyr128 – Pi-Pi Tyr126 – Pi-donor H-bond Tyr126 – Pi-Pi Glu123 – H-bond FAD – Pi-Pi	4/1	6	3.61	0	0	No; MW<250, XLOGP3>3.5
**BHT**	−6.0	Tyr128 – Pi-Pi Tyr128 – Pi-alkyl Tyr128 – Pi-sigma Tyr126 – Pi-alkyl Gly149 - Carbon H-bond FAD – Pi-sigma FAD – Pi-alkyl	1/1	2	4.24	0	2; XLOGP3>5, Heteroatoms<2	No; MW<250, XLOGP3>3.5
